# Circulating MicroRNAs Expression Profile in Lung Inflammation: A Preliminary Study

**DOI:** 10.3390/jcm11185446

**Published:** 2022-09-16

**Authors:** Davida Mirra, Erika Cione, Giuseppe Spaziano, Renata Esposito, Mario Sorgenti, Elisabetta Granato, Ida Cerqua, Lucia Muraca, Pasquale Iovino, Luca Gallelli, Bruno D’Agostino

**Affiliations:** 1 Department of Environmental Biological and Pharmaceutical Sciences and Technologies University of Campania “Luigi Vanvitelli”, 81100 Caserta, Italy; 2 Department of Pharmacy, Health and Nutritional Sciences-Department of Excellence 2018–2022, University of Calabria, 87036 Rende, CS, Italy; 3 Respiratory Diseases in Primary Care, ASP Catanzaro, 88100 Catanzaro, Italy; 4 Department of Pharmacy, School of Medicine and Surgery, University of Naples Federico II, 80138 Naples, Italy; 5 Department of Primary Care, ASP Catanzaro, 88100 Catanzaro, Italy; 6 Clinical Pharmacology and Pharmacovigilance Unit, Department of Health Sciences, Mater Domini Hospital, University of Catanzaro, 88100 Catanzaro, Italy

**Keywords:** circulating microRNAs, asthma, biomarkers, inflammation

## Abstract

Background: Bronchial asthma is an inflammatory airway disease with an ever-increasing incidence. Therefore, innovative management strategies are urgently needed. MicroRNAs are small molecules that play a key role in lungs cellular functions and are involved in chronic inflammatory diseases, such as bronchial asthma. This study aims to compare microRNA serum expression between subjects with asthma, obesity, the most common co-morbidity in asthma, and healthy controls to obtain a specific expression profile specifically related to lung inflammation. Methods: We collected serum samples from a prospective cohort of 25 sex-matched subjects to determine circulating miRNAs through a quantitative RT-PCR. Moreover, we performed an in silico prediction of microRNA target genes linked to lung inflammation. Results: Asthmatic patients had a significant lower expression of hsa-miR-34a-5p, 181a-5p and 146a-5p compared to both obese and healthy ones suggesting microRNAs’ specific involvement in the regulation of lungs inflammatory response. Indeed, using in silico analysis, we identified microRNAs novel target genes as GATA family, linked to the inflammatory-related pathway. Conclusions: This study identifies a novel circulating miRNAs expression profile with promising potentials for asthma clinical evaluations and management. Further and larger investigations will be needed to confirm the potential role of microRNA as a clinical marker of bronchial asthma and eventually of pharmacological treatment response.

## 1. Introduction

Bronchial asthma is a chronic inflammatory airway disease characterized by an abnormal imbalance between Th1 cells, which secrete IL-2 and IFN-γ, and Th2 cells that produce IL-4, IL-5, IL-6 and IL-13, involved in both inflammation and allergic response [1,2,3,4,5]. It is often caused by chronic exposition to allergens or correlated to other diseases [6,7,8,9]. Even if international guidelines suggest a step-by-step treatment of bronchial asthma, to date, the treatment is not completely effective [10,11]. Indeed, bronchial asthma is a heterogeneous disease associated with several endotypes or comorbidities among which obesity has recently been identified [12]. Obesity is the most common asthma co-morbidity, characterized by an increase in the number of adipocytes and fat stored in the cells [13,14,15,16]. The immune cell composition of adipose tissue undergoes multiple changes during the development of obesity [17] with abnormal cytokine and chemokine production and activation of inflammatory signaling pathways [18]. Furthermore, obese patients show a certain state of chronic low-grade inflammation, which can be detected to some degree in the blood [19]. However, although obesity-related systemic inflammation and bronchial asthma share some similarities, they have fundamental differences such as clinical features, prognosis, and pathophysiology, which are not yet well understood [20]. In this context, identifying specific biomarkers is essential to help characterize and clinically classify bronchial asthma for early diagnosis and eventually for pharmacological treatment response. Previously, we documented that microRNAs (miRs) are involved in several inflammatory diseases [21,22,23,24]. miRs are small non-coding RNA typically consisting of approximately 18–22 nucleotides which control gene expression by acting as post-transcriptional regulators and silencing their downstream target mRNA [25]. Emerging studies demonstrate the role of miRs in the regulation of physiological intracellular functions highlighting their ability to influence cell differentiation and proliferation, immune activation and inflammatory responses by activation of intracellular signaling pathways [26]. Moreover, it has been documented that asthma could be under the control of several miRs as demonstrated by noticeable changes in gene expression and protein synthesis in the airways [27]. Indeed, dysregulated levels of several miRs, even in nasal biopsies of asthmatic patients, without differences between allergic and non-allergic phenotypes were reported [28,29,30,31].

Notably, miR-146a is upregulated in asthmatics serum and related to impaired asthma control. miR-21 is involved in asthma pathogenesis by promoting Th2 activation and regulating eosinophil growth. miR-223 is able to influence the proliferation of eosinophil progenitors. Moreover, miR-155 has recently emerged as a modulator in inflammation-associated asthma and allergy. Therefore, the current literature data shed light on the importance of analyzing the abovementioned miRNA for a better characterization of asthma and lung inflammation, in general [32].

However, even though several miRNAs are critically involved in asthma, a specific miRNA signature for bronchial asthma is still needed because circulating miRNA profiles, linked to inflammation, are common in obesity and in asthma [33].

Indeed, some single miRNAs or, more probably, their sets hold the promise for their use as biomarkers of lung inflammatory diseases and target of future therapies.

The difficulty to obtain tissue biopsies prompted the study of circulating miRs because they do not require invasive procedures and maintain the biochemical characteristics of the intracellular tissue counterpart. The circulating miRs, released by asthma-related cells into extracellular vesicles as exosomes, participate to the intracellular communication and crosstalk by acting as mediators of information exchange, thus contributing to airway hyper-responsiveness, airway inflammation and airway remodeling [34,35]. Because circulating miRNA profiles have recently been reported as an attractive tool for improving the diagnosis of diseases, including life-threatening pathologies through liquid biopsy, here, we performed an analysis of a miRNA panel in subjects with obesity, healthy ones and in asthmatic treated patients to obtain a specific expression profile related to lung inflammation.

## 2. Materials and Methods

### 2.1. Population

We performed an observational clinical study on 25 age- and sex-matched subjects with asthma or obesity and healthy controls recruited from the “Mater Domini” Hospital in Catanzaro, Italy. All patients underwent routine peripheral blood sampling according to normal clinical practice and were selected based on their clinical features. This study is part of the clinical trial recorded in clinicaltrials.gov (NCT04567212) and approved by the local Ethics Committee “Calabria Centro”. This work was conducted in compliance with the Institutional Review Board/Human Subjects Research Committee requirements and the Declaration of Helsinki and the Guidelines for Good Clinical Practice criteria. Before the beginning of the study, all the enrolled patients or legal guardians signed the informed consent.

Inclusion criteria: Patients of both sexes, aged >6-year and <85-year, with symptoms of bronchial asthma in agreement with international guidelines [36] or not asthmatics obese with Body Mass Index (kg/m^2^) higher than 30 not receiving a bronchodilator treatment or immunosuppressive or anti-histaminergic drugs.

Exclusion criteria: We excluded all subjects with severe asthma or with mixed asthma (asthma and chronic obstructive pulmonary disease); patients with active pulmonary infections or who had taken anti-obesity drugs within the last 6 months, and subjects with metabolic syndrome, arthritis or immune disorders. In addition, those who did not sign the informed consent were excluded from participating.

Endpoint: The statistically significant difference (*p* < 0.05) of miRs expression in moderate asthmatic norm-weight patients (MANW) in respect to healthy no asthmatics obese subjects (HNAO) with systemic inflammation and healthy norm-weight ones (HNW).

In agreement with the criteria of recruitment, we enrolled 10 healthy and norm-weight patients (4 women mean age 48.8 ± 13.1 and 6 men, mean age 41.3 ± 9.0) as control group (HNW, Group A). Moreover, we evaluated 42 asthmatics patients and after clinical and functional evaluation, 10 of these (26.2%) (5 women and 5 men; mean age 38.9 ± 19.7) were enrolled (MANW, Group B) and signed the informed consent. We excluded 32 patients because 16 presented severe asthma and were in treatment with monoclonal antibody, 12 presented a mixed asthma (asthma/COPD) and 4 refused to sign the consent form. A last group of 5 patients (3 women: mean age 52.3 ± 19.6 and 2 men, mean age 40.5 ± 10.6) healthy not asthmatics obese with systemic inflammation (HNAO, Group C) were enrolled.

### 2.2. Data Collection and Clinical Biochemistry Assays

Clinical characteristics and treatment data were obtained at the time of enrollment and were reviewed by a trained team of physicians. Blood samples were taken at the time of enrollment and were stored at −80 °C for the other evaluations and were prepared in agreement with our previous paper [24]. Total RNA was extracted from 200 μL of blood using miRNeasy Serum/Plasma Kit (QIAGEN), in order to lower possible contaminants [23,37]. The total extracted miRs were quantified with a qubit microRNA Assay Kit that allows easy and accurate quantification of miRs using the Qubit 4 Fluorometer. Although the reagent is not exclusively selective for miRs, it can reproducibly quantify miRs in pure samples at levels low than 0.5 ng even in the presence of other RNAs including mRNA. A volume of 1 µL of each sample was quantified.

### 2.3. Quantitative Real Time PCR (qRT-PCR)

The quantification of miRs was performed with a method termed looped primer RT-PCR, following the Thermo Fisher Scientific (Waltham, MA, USA) [38]. Briefly, 5–10 ng of total RNA was subjected to reverse transcription polymerase chain reaction using the TaqMan MicroRNA Reverse Transcription kit for all the miR targets chosen, according to a manufacturer’s protocol. The thermocycling conditions were: 30 min at 42 °C, 5 min at 85 °C and 5 min at 4 °C.

Then, qRT-PCR was performed using TaqMan Universal PCR Master Mix Kit (Thermo Fisher Scientific, Waltham, MA, USA) according to the manufacturer’s protocol and the equipment QuantumStudio3TM Real-Time PCR Systems [22]. The thermocycling conditions were: 95 °C for 10 min and 40 cycles of 15 s at 95 °C, followed by 1 min at 60 °C. After finalization of the qRT-PCR experiments, the cycle threshold (Ct) of the reactions were determined. The delta Ct method was adopted, and the difference was calculated and plotted as follows: Ct total cycles 40—Ct target. The difference was plotted as Δct directly as previously described [39].

### 2.4. In Silico Prediction of Hsa-miRs Target Genes

In order to identify genes as target of hsa-miR-34a-5p, 181a-5p, 150-5p, 21-5p, 155-5p, 223-5p, 125b-5p and 146a-5p linked to allergy and lung function, we performed in silico analysis.

### 2.5. Statistical Analysis

All data are expressed as mean ± standard deviation (SD). We used both nominal (gender, co-morbidity and treatment) and categorical (age, weight and grade of disease) variables. The one-way ANOVA test was used to evaluate the differences between the groups. Differences identified by ANOVA were examined using Kruskal–Wallis test followed by Dunn’s Multiple Comparison Test. The Pearson test was used to evaluate the correlation between microRNAs expression and clinical characteristics (e.g., age and gender). GraphPad 8.0 software was used for the statistical analyses (GraphPad Software, San Diego, CA, USA). The differences were considered significant for values of *p* < 0.05.

## 3. Results

### 3.1. Patients

Clinical characteristics and pharmacological treatment are reported in Table 1 and Table 2, respectively. History revealed a significant difference (*p* < 0.01) in the age between women (mean age 43.8 ± 19.3) and men (mean age 33 ± 20.8) even if we failed to report a difference in comorbidity between these groups. Amongst the groups, we did not find differences with respect to age (*p* = Group A vs. B: 0.44; Group A vs. C: 0.70; Group B vs. C: 0.38) or comorbidity.

### 3.2. miRs Expression in Asthmatics

**Figure 1:**
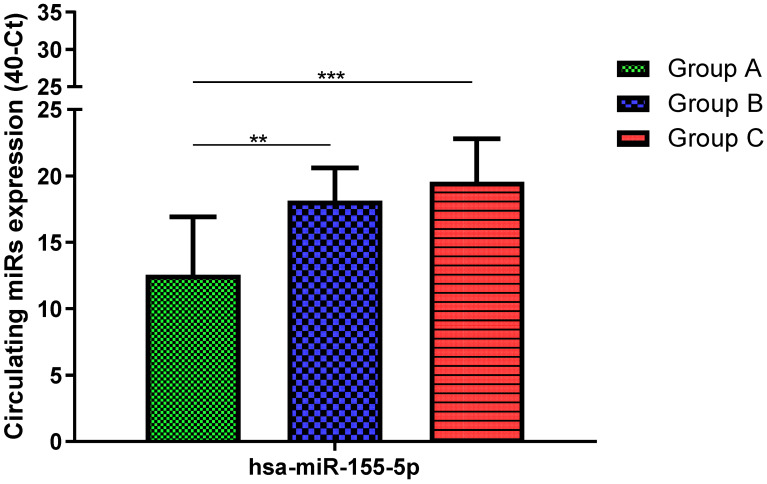
Serum levels of hsa-miR-155-5p in HNW (Group A), MANW (Group B) and HNAO (Group C). Results were shown as means ± SD. The statistical tests used in these analyses were one-way analysis of variance using Kruskal–Wallis test followed by Dunn’s Multiple Comparison Test. ** *p* < 0.01, *** *p* < 0.001.

**Figure 2:**
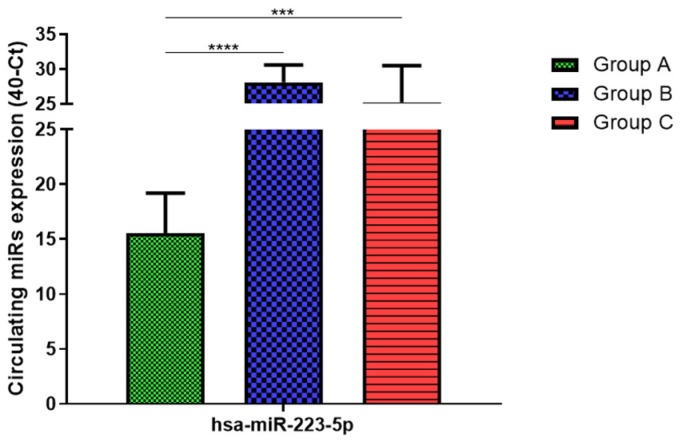
Serum levels of hsa-miR-223-5p in HNW (Group A), MANW (Group B) and HNAO (Group C). Results were shown as means ± SD. The statistical tests used in these analyses were one-way analysis of variance using Kruskal–Wallis test followed by Dunn’s Multiple Comparison Test. *** *p* < 0.001, **** *p* < 0.0001.

**Figure 3:**
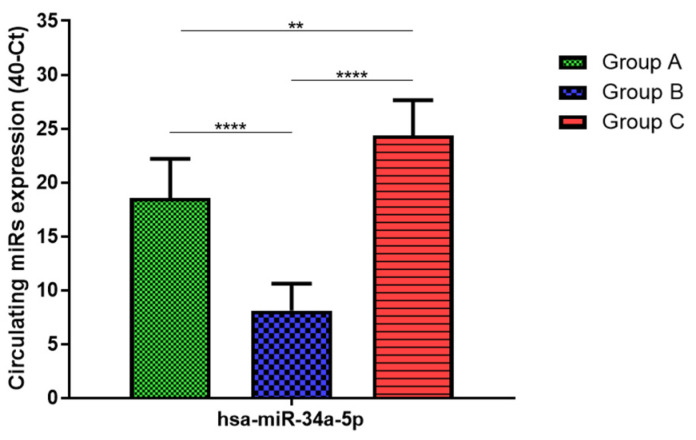
Serum levels of hsa-miR-34a-5p in HNW (Group A), MANW (Group B) and HNAO (Group C). Results were shown as means ± SD. The statistical tests used in these analyses were one-way analysis of variance using Kruskal–Wallis test followed by Dunn’s Multiple Comparison Test. ** *p* < 0.01, **** *p* < 0.0001.

**Figure 4:**
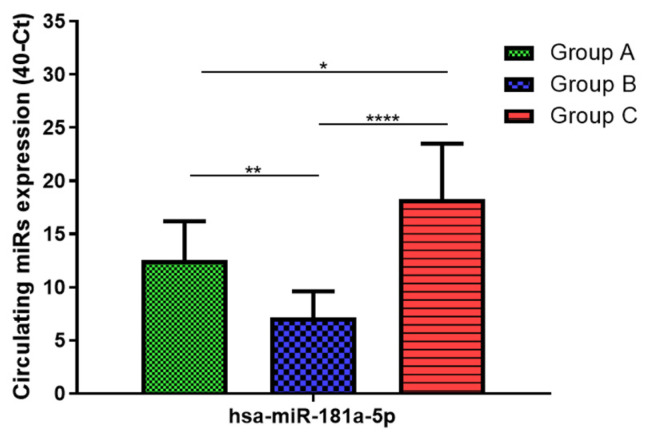
Serum levels of hsa-miR-181a-5p in HNW (Group A), MANW (Group B) and HNAO (Group C). Results were shown as means ± SD. The statistical tests used in these analyses were one-way analysis of variance using Kruskal–Wallis test followed by Dunn’s Multiple Comparison Test. * *p* < 0.05, ** *p* < 0.01, **** *p* < 0.0001.

**Figure 5:**
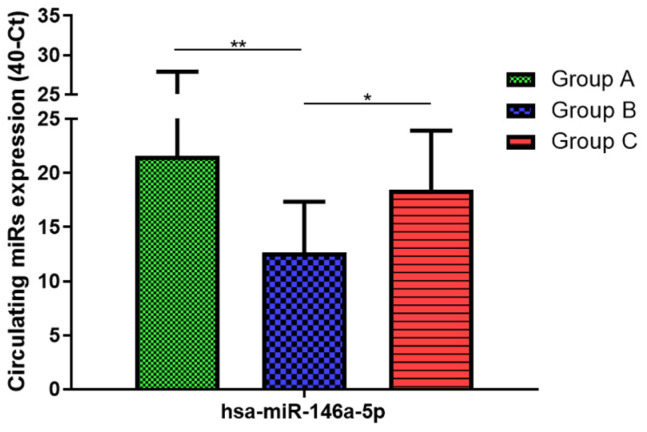
Serum levels of hsa-miR-146a-5p in HNW (Group A), MANW (Group B) and HNAO (Group C). Results were shown as means ± SD. The statistical tests used in these analyses were one-way analysis of variance using Kruskal–Wallis test followed by Dunn’s Multiple Comparison Test. * *p* < 0.05, ** *p* < 0.01.

**Figure 6:**
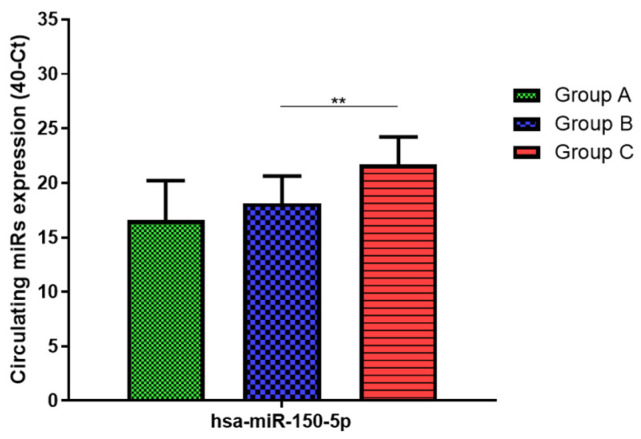
Serum levels of hsa-miR-150-5p in HNW (Group A), MANW (Group B) and HNAO (Group C). Results were shown as means ± SD. The statistical tests used in these analyses were one-way analysis of variance using Kruskal–Wallis test followed by Dunn’s Multiple Comparison Test. ** *p* < 0.01.

**Figure 7:**
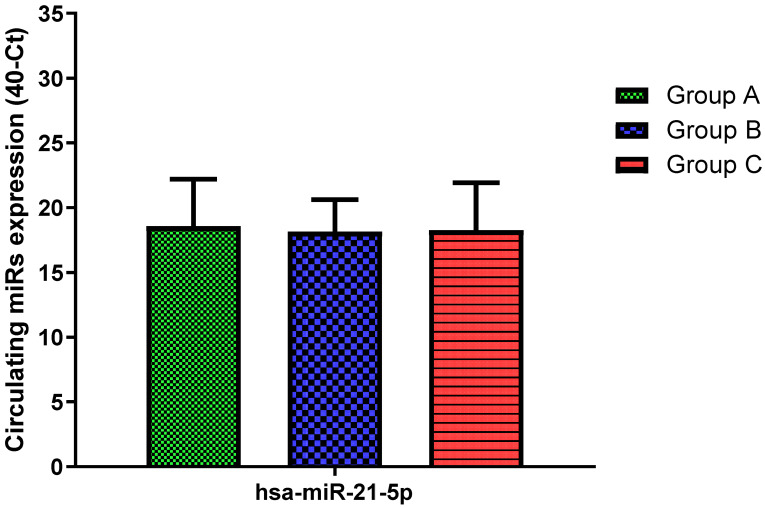
Serum levels of hsa-miR-21-5p in HNW (Group A), MANW (Group B) and HNAO (Group C). Results were shown as means ± SD. The statistical tests used in these analyses were one-way analysis of variance using Kruskal–Wallis test followed by Dunn’s Multiple Comparison Test.

**Figure 8:**
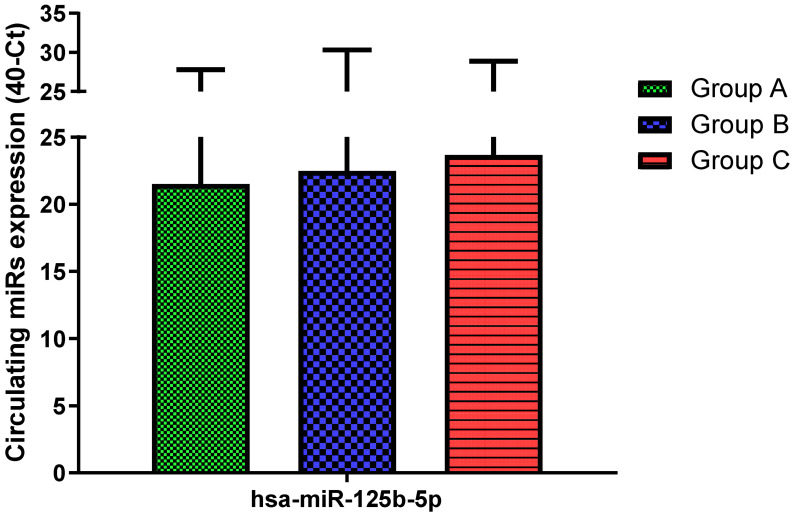
Serum levels of hsa-miR-125b-5p in HNW (Group A), MANW (Group B) and HNAO (Group C). Results were shown as means ± SD. The statistical tests used in these analyses were one-way analysis of variance using Kruskal–Wallis test followed by Dunn’s Multiple Comparison Test.

Total RNA was extracted from serum frozen fractions with an extraction efficiency between 82 and 97% for all samples analyzed (Supplementary Table S2). The hsa-miR-155-5p and 223-5p underwent significant positive modulation in MANW compared to HNW with a *p*-value of <0.01 and ˂0.0001, respectively (Figure 1 and Figure 2). qRT-PCR analysis indicated that the hsa-miR-34a-5p, 181a-5p and 146a-5p were significantly reduced in MANW compared to HNW with a *p*-value of ˂0.0001, <0.01 and <0.01, respectively (Figure 3, Figure 4 and Figure 5). While no significant difference was recorded for hsa-miR-150-5p, 21-5p and 125b-5p (Figure 6, Figure 7 and Figure 8).

### 3.3. miRs Expression in Obese

qRT-PCR analysis indicated that the hsa-miR-155-5p, 223-5p, 34a-5p and 181a-5p, were significantly increased in HNAO compared to HNW with a *p*-value of <0.001, <0.001, <0.01 and <0.05, respectively (Figure 1, Figure 2, Figure 3 and Figure 4). While no significant difference was recorded for hsa-miR-146a-5p, 150-5p, 21-5p and 125b-5p (Figure 5, Figure 6, Figure 7 and Figure 8).

### 3.4. miRs Expression in Asthmatics vs. Obese

Determination via qRT-PCR analysis indicated that the hsa-miR-34a-5p, 181a-5p, 146a-5p and 150-5p were significantly lower in MANW compared to HNAO, with a *p*-value of ˂0.0001 for the first two and of <0.05 and <0.01 for the latter two (Figure 3, Figure 4, Figure 5 and Figure 6). Meanwhile, no significant difference was recorded for and hsa-miR-155-5p, 223-5p, 21-5p and 125b-5p (Figure 1, Figure 2, Figure 7 and Figure 8).

### 3.5. In Silico Results

Two different databases were used for the in silico analysis. Data were compared with respect to the number of target genes experimentally validated in both databases. The results are reported in Table 3. In DIANA tools, the numbers of validated target genes were higher with respect to the miR target Link Human; therefore, DIANA tools were used for bioinformatics analysis. Predicted and validated target genes were assessed using DIANA Tools. The hsa-miR-21-5p, hsa-miR-181a-5p and hsa-miR-34a-5p were found to regulate GATA Zinc Finger Domain Containing 2B (GATAD2B), GATA Binding Protein 6 (GATA6) and GATA Binding Protein 3 (GATA3), involved in T-cell development, as shown in Table 4. The hsa-miR-34a-5p was found to regulate IL17RB, IL6R and IL2RB, IL6R, and IL9R, which are, respectively, involved in the NF-κB pathway, T cell-mediated immune responses, CCRGC signaling pathways and AKT signaling pathway. However, other target genes were found to be influenced by hsa-miR-155-5p, hsa-miR-150-5p, hsa-miR-223-5p and hsa-miR-146a-5p and linked to an inflammatory-related pathway such as cytokine production, maturation and activation of the immune cells, as shown in Table 4. Abbreviations and gene names are described in Table 5.

## 4. Discussion

In this study, we identified circulating similarities and differences in the miRs expression profile of moderate asthmatic patients compared with obese and healthy controls. Bronchial asthma is a chronic inflammatory disease of the lower airways in which many cells play a key role: in particular, mast cells, eosinophils, T lymphocytes, macrophages, neutrophils, and epithelial cells [1]. Recent studies highlighted that different asthma endotypes are related to metabolic traits, such as obesity [40]. Indeed, obesity is characterized by a state of chronic inflammation and abnormal synthesis of cytokines leading to an imbalance of anti-inflammatory biochemical pathways [21]. Both diseases are heritable traits, and the parallel rise in their prevalence worldwide suggests these conditions could have common genetic and environmental risk factors [41]. However, although obesity-related systemic inflammation and bronchial asthma shares some similarities, they have fundamental differences such as clinical features, prognosis and pathophysiology, which are not yet well understood [20]. The co-morbidity of obesity in certain patients with asthma has recently been identified as a unique phenotype. On the other hand, obese patients show a certain type of subclinical chronic inflammation, which can be misleading [15,16,33]. Therefore, we performed an analysis of a miRs panel to obtain a specific expression profile more related to lung inflammation than to systemic inflammation. We found a common hsa-miR-155-5p and 223-5p trend in moderate asthmatics and obese with an increase in both miRs levels compared to healthy controls. These findings were in accordance with Daniel et al. [42], who showed that hsa-miR-155-5p expression is increased in CD4+ T cells of asthmatics compared to non-asthmatics exposed to dust mites and is positively associated with the Th2 cytokine profile. Karam and colleagues [43] showed that plasma hsa-miR-155-5p was correlated positively with Il-13 levels and correlated negatively with FEV1and FVC. In addition, hsa-miR-155-5p deficiency led to a decrease in eosinophilic inflammation in sensitized allergic mice compared to control animals [44]. It is well known that an imbalance of Th1/Th2 cell and abnormal Th17 cell immunity plays a vital role in the pathogenesis of asthma exacerbating airway inflammation by Th2 cytokines secretion [45]. Similarly, Th17 cells can lead to severe airway inflammation by IL-17A secretion [46]. Xu et al. [47] reported that there was a positive correlation between leukocytes miR-223-3p and IL-17A levels in asthmatics, which suggested that the upregulation of miR-223-3p levels may play a key role in airway inflammation. Interestingly, both miRs were also upregulated in the obese suggesting that their expression may be index of a common inflammation profile with bronchial asthma. Indeed, hsa-miR-155-5p is one of the miRNAs that has been reported to be overexpressed in obese adipose tissue macrophages exosomes [48], while higher levels of hsa-miR-223-5p were found in obese adolescents compared to normal-weight ones [49]. Despite this similar miRs expression pattern, bronchial asthma and obesity showed opposite trends in hsa-miR-34a-5p, 181a-5p and 146a-5p. Adipocytes transmitted signals of nutrient overload to the adipose-resident macrophages, mediating exacerbation of obesity-induced systemic inflammation and metabolic dysregulation, which were reported to be mediated by exosomes secreted hsa-miR-34a-5p [50]. Consistent with these data, we observed a significant increase in hsa-miR-34a-5p in obese with respect to both asthmatic patients and healthy ones. Instead, we found an inverse hsa-miR-34a-5p expression in patients with moderate asthma. Moreover, hsa-miR-34a-5p was found down-regulated in exosomes derived from the bronchial epithelial cells stimulated by inflammatory factors [51]. In addition, Ding et al. [52] indicated that hsa-miR-34a-5p suppressed proliferation and migration in an in vitro model of airway smooth muscle cells, a key process of airway remodeling in asthma. Similarly, hsa-miR-181a-5p trended oppositely between asthmatics and obese, suggesting a decrease in asthma and an increase in obesity, despite mixed literature data [53,54,55]. T cells are key regulators of the development and maintenance of the inflammatory response in asthma; specifically, severe asthma was associated with the activation of circulating CD8+ T cells, and this response is correlated with the downregulation of hsa-miR-146a [56]. In accordance, our data showed evidence that asthma status was associated with a significant decrease of hsa-miR-146a compared to both healthy and obese subjects suggesting its specific role in lung inflammation. Obesity is mainly due to the intricately intertwined crosstalk of various pro- and anti-inflammatory signaling pathways involved in the immune response, in which B cells play a central role [57]. In this context, down-regulation of has-miR-150-5p has been found to modulate adipose tissue function by controlling B-cell activation and their interactions with other immune cells [58]. Consistently, we found higher has-miR-150-5p expression in obese patients although it reached statistical significance only versus moderate asthmatics, suggesting a specific obesity signature. Finally, no significant change has been seen in the expression of hsa-miR-21-5p and 125b-5p in all groups despite previous studies showing that both could be dysregulated in asthma [59,60]. In order to underline miRs role in asthma, we performed an in silico analysis identifying target genes associated with asthma. Amongst them, the phosphodiesterase (PDE) captured our interest. PDE enzymes hydrolyze cAMP, which is a second messenger and a mediator of many cellular signals. Thus, by regulating cAMP cellular concentration, this protein plays a key role both in physiological processes and diseases. Indeed, cAMP mediates relaxation of airway smooth muscle so PDE4 inhibitors are currently available for asthma treatment [61,62]. Moreover, PDE, particularly PDE4, regulate inflammatory and structural cells. Interestingly, we found an in silico interaction between hsa-miR-181a-5p and PDE8B and between hsa-miR-34a-5p and PDE7A and PDE4B genes. These genes encode for proteins that belong to PDE family. Since hsa-miR181a-5p and 34a-5p were both downregulated in MANW, this dysregulation may lead to an aberrant expression of PDE with smooth muscle contraction and lung function reduction. Several cytokines contribute to asthma manifestations. IL-6 is a cytokine that regulates cell growth and differentiation, immune response and its expression was related to metabolic dysfunction and asthma severity [63], IL-9 orchestrate inflammation that takes place in asthma [64] while IL2RB is involved in T cell-mediated immune responses [65]. Considering that hsa-miR34a-5p targets IL-9, IL-6 and IL2RB genes, its dysregulation could be an indication of the aberrant expression of these cytokines in MANW. T-cell development and IL-4, IL-5, and IL-13 secretion from Th2 cells in asthmatics were regulated by many transcription factors such as GATA3 [66] while GATA6 regulates lung epithelium development [67]. Our findings showed that there were interactions between hsa-miR-181a-5p and GATA6 gene and between hsa-miR-34a-5p and GATA3 gene, suggesting that their downregulation in MANW leads to increased expression of these genes and changes in lung homoeostasis.

## 5. Conclusions

Herein, we presented a pilot study to analyze serum miRs profiles related to lung inflammation. The limitation of this study is the small sample size for miRs analyses. Nevertheless, we provide the preliminary data for future studies involving a larger population.

Taken together, these data, even if preliminary, highlighted that circulating miRs expression levels not only are linked to inflammation status but may represent a specific signature of lung inflammation, suggesting their possible role as biomarkers for improving diagnosis and clinically classifying bronchial asthma. Moreover, circulating miRs might be modulated by drugs, pointing to their expression levels as an index of therapy response. Therefore, further investigations in a larger cohort are needed to clarify miRs signature in asthma and how drug modulation of miRs can lead to a significant alternation of mRNA targets and their biological functions.

## Figures and Tables

**Table 1:** Demographic characteristics.

Characteristics	HNW Group A	MANW Group B	HNAO Group C
Men	6	5	3
Women	4	5	2
Mean Age	44.3 ± 10.8	38.9 ± 19.7	47.6 ± 16.2
Allergy	0	6	0
Moderate Asthma	0	10	0
Blood hypertension	3	4	3
Smoke	6	7	1
Diabetes type 1	2	1	1
Diabetes type 2	1	1	1
Obesity	0	0	5
BMI	25.36 ± 2.06	28.51 ± 3.25	47.53 ± 4.42
Waistline	69.62 ± 2.81	81.23 ± 4.18	137 ± 6.69
Hips	100.9 ± 7.78	102.5 ± 2.83	141 ± 13.22

**Table 2:** Pharmacological treatments of enrolled asthmatics Group B MANW.

Patients Group B MANW #	Gender	Ebastine	Formoterol	Salbutamol	Beclomethasone
**1**	**W**	**X**	**X**		
**2**	**W**	**X**	**X**		
**3**	**M**	**X**	**X**		
**4**	**M**	**X**	**X**		
**5**	**M**	**X**	**X**		
**6**	**W**	**X**			
**7**	**W**	**X**			
**8**	**M**	**X**			
**9**	**W**	**X**		**X**	
**10**	**M**	**X**	**X**		**X**

**Table 3:** Bioinformatics tools for in silico analysis.

Number of Target Genes		
miR	miR Target Link Human	DIANA Tools
hsa-miR-34a-5p	864	1108
hsa-miR-181a-5p	596	560
hsa-miR-150-5p	632	1119
hsa-miR-21-5p	588	521
hsa-miR-155-5p	918	1084
hsa-miR-223-5p	271	397
hsa-miR-125b-5p	522	1085
hsa-miR-146a-5p	260	853

**Table 4:** Biochemical pathways and possible miRs gene interaction.

Biochemical Pathways			
**T-cell** **development**	**miR**	**Validated target genes**	**Asthma phenotypes**
	hsa-miR-21-5p	GATAD2B	Atopic asthma
	hsa-miR-181a-5p	GATA6	extrinsic asthma with acute exacerbation
	hsa-miR-34a-5p	GATA3	extrinsic asthma with status asthmaticus extrinsic asthma asthma allergic atopic asthma, susceptibility to IgE-mediated allergic asthma
**NF-kappa B** **pathway;** **T cell-mediated immune** **responses;** **CCRGC Signaling Pathways;** **AKT Signaling Pathway**	hsa-miR-223-5p	IL17R	Asthma, susceptibility to asthma, bronchial asthma-related traits asthma, nocturnal chronic obstructive asthma chronic obstructive asthma with acute exacerbation chronic obstructive asthma with status asthmaticus exercise-induced asthma
	hsa-miR-34a-5p	IL2RB, IL6R, IL9R	
**Cyclic nucleotides signal** **transduction.**	hsa-miR-181a-5p	PDE8B	acute and chronic respiratory failure
	hsa-miR-150-5p	PDE7A	acute respiratory failure
	hsa-miR-34a-5p	PDE7A, PDE4B	acute-on-chronic respiratory failure chronic respiratory failure respiratory insufficiency/failure pulmonary valve insufficiency respiratory insufficiency chronic respiratory disease chronic disease of respiratory system respiration disorders respiratory tract diseases
**SMAD2/SMAD3-SMAD4 pathway.**	hsa-miR-150-5p	SP1	asthma, susceptibility to asthma, bronchial asthma-related traits asthma, nocturnal, asthma, diminished response to antileukotriene treatment in bronchial hyperreactivity chronic obstructive asthma chronic obstructive asthma with acute exacerbation chronic obstructive asthma with status asthmaticus exercise induced asthma
**Increased cytokines production IL33, IL10**	hsa-miR-155-5p	SOCS1	
**Inhibits degranulation and IL-12 production**	hsa-miR-21-5p	IL-12p35	
		P38	
	hsa-miR-146a-5p	IRAK1	
**Negative regulator of NFkB activation**	has-miR-125b-5p		

**Table 5:** Abbreviations, gene names, methods and tissue of validation.

Abbreviation	Gene Name	Methods	Tissue
**GATAD2B**	GATA Zinc Finger Domain Containing 2B	IP	Kidney, Mammary Gland
**GATA6**	GATA Binding Protein 6	MA, IP	Kidney
**GATA3**	GATA Binding Protein 3	IP, Bi	Mammary Gland, Intestine
**IL17RB**	Interleukin 17 Receptor B	RA	Bone Marrow
**IL6R**	Interleukin 6 Receptor	Qp	Peripheral Blood
**IL2RB**	Interleukin 2 Receptor Subunit Beta	IP	Bone Marrow
**IL9R**	Interleukin 9 Receptor	Bi	Intestine
**PDE8B**	Phosphodiesterase 8B	IP	Kidney
**PDE7A**	Phosphodiesterase 7A	IP	Bone Marrow
**PDE4B**	Phosphodiesterase 4B	Bi, IP	Intestine
**SP1**	Sp1 Transcription Factor	IP	Pancreas
**SOCS1**	Suppressor Of Cytokine Signaling 1	RA, Qp, WB	Bone Marrow, Adipose
**IL12p35**	Interleukin 12	N/A	N/A
**IRAK1**	Interleukin 1 Receptor Associated Kinase 1	IP, WB	N/A

## Supplementary Materials

The following supporting information can be downloaded at: https://www.mdpi.com/article/10.3390/jcm11185446/s1; Table S1: miRs are up- and down-regulated in PBMCs of asthmatic subjects compared to healthy controls; Table S2: % Extraction efficiency.
