# Supplementary material for: Circulating MicroRNAs Expression Profile in Lung Inflammation: A Preliminary Study
Source: J Clin Med. 2022 Sep 16;11(18):5446. doi: 10.3390/jcm11185446 (PMC9500709; doi:10.3390/jcm11185446)
Supplement: Supplementary file 1 [file jcm-11-05446-s001.zip › jcm-1863206-supplementary.pdf]

Supplemental data.

1.1 Population

We performed an observational clinical study on 25 age- and sex-matched subjects with asthma or obesity and healthy controls recruited from the "Mater Domini" Hospital in Catanzaro, Italy. All patients underwent routine peripheral blood sampling according to normal clinical practice and were selected based on their clinical features. This study is a part of the clinical trial recorded in clinicaltrials.gov (NCT04567212) and approved by the local Ethics Committee “Calabria Centro”. This work was conducted in compliance with the Institutional Review Board/Human Subjects Research Committee requirements and the Declaration of Helsinki and the Guidelines for Good Clinical Practice criteria. Before the beginning of the study, all the enrolled patients or legal guardians signed the informed consent.

Inclusion criteria: Patients of both sexes, aged > 6-year and <85-year, with symptoms of bronchial asthma in agreement with international guidelines [31] or not asthmatics obese with Body Mass Index (kg/m^2) higher than 30 not receiving a bronchodilator treatment or immunosuppressive or anti-histaminergic drugs.

Exclusion criteria: We excluded all subjects with severe asthma or with mixed asthma (asthma and chronic obstructive pulmonary disease), patients with active pulmonary infections or who had taken anti-obesity drugs within the last 6 months, subjects with metabolic syndrome, arthritis, or immune disorders. In addition, those who did not sign the informed consent were excluded from participating.

Endpoint: The statistically significant difference ($P<0.05$) of miRs expression in moderate asthmatic norm-weight patients (MANW) in respect to healthy no asthmatics obese subjects (HNAO) with systemic inflammation and healthy norm-weight ones (HNW).

In agreement with the criteria of recruitment, we enrolled 10 healthy and norm-weight patients (4 women mean age 48.8 ± 13.1 and 6 men, mean age 41.3 ± 9.0) as control group (HNW, Group A).

Moreover, we evaluated 42 asthmatics patients and after clinical and functional evaluation, 10 of these (26.2 %) (5 women and 5 men; mean age 38.9 ± 19.7) were enrolled (MANW, Group B) and signed the informed consent. We excluded 32 patients because 16 presented severe asthma and were in treatment with monoclonal antibody, 12 presented a mixed asthma (asthma/COPD) and 4 because refused to sign the consent form. A last group of 5 patients (3 women: mean age 52.3 ± 19.6 and 2 men, mean age 40.5 ± 10.6) healthy not asthmatics obese with systemic inflammation (HNAO, Group C) were enrolled.

1.2 Data Collection and Clinical Biochemistry Assays

Clinical characteristics and treatment data were obtained at the time of enrollment and were reviewed by a trained team of physicians. Blood sample was taken at the time of enrollment and was stored to -80°C for the other evaluations. Samples of blood plasma were prepared in agreement with our previous paper. Briefly, venous blood samples were drawn after a 12 h overnight fast and processed within 2 h from collection. Serum for miRNAs analysis was separated by centrifugation at 1800 x g for 10 min at room temperature, collected in RNase-free tubes and further centrifuged at 1200 x g for 20 min at 10 °C to completely remove contaminant cells. Finally, serum samples were divided in aliquots to avoid freeze–thaw cycles and finally stored at −80 °C up to the RNA extraction.

1.3 Total RNAs extraction

Total RNA was extracted from 200 µl of blood by using miRNeasy Serum/Plasma Kit (QIAGEN), in order to lower possible contaminants. Briefly, samples were lysed with 5 volumes of QIAzol Lysis Reagent for 5 min at room temperature. Five µl of exogenous ath-miR-159a (1 nM) from Arabidopsis thaliana spike-in control (Diatech Lab Line, Jesi (AN) Italy) and 200 µl of chloroform were added. The mixture was vortexed, kept at room temperature for 5 min, centrifuged at 12000 x g for 15 min and the upper (aqueous) phase was collected. Subsequently, 1.5 volumes of 100% ethanol were added

to the aqueous phase. The obtained solution was passed through a RNeasy MinElute spin column in sequential 700 µl aliquots, where the total RNA binds to the membrane and phenol and other contaminants were efficiently washed away using specific buffers. Finally, RNA was washed once with 80% ethanol, dried for 2 minutes by centrifugation, and dissolved in 15 µl of RNase-free water.

1.4 Total microRNAs quantification

The total extracted miRs were quantified by qubit microRNA Assay Kit that allows easy and accurate quantification of miRs using the Qubit 4 Fluorometer. Although the reagent is not exclusively selective for miRs, it can reproducibly quantify miRs in pure samples at levels low than 0.5 ng even in the presence of other RNAs including mRNA. 1 µL of each sample was quantified.

1.5 MicroRNAs Loop Primer Method

The quantification of miRNA was performed with a method termed looped primer RT-PCR, following the Thermo Fisher Scientific (Waltham, Massachusetts, USA) protocol. Initially, 5-10 ng of total RNA was subjected to reverse transcription polymerase chain reaction using the TaqMan MicroRNA Reverse Transcription kit (Thermo Fisher Scientific, Waltham, Massachusetts, USA) for all the miR targets chosen, according to manufacturer's protocol. The thermocycling conditions were: 30 min at 42 °C, 5 min at 85 °C and 5 min at 4 °C. The reactions were performed for each patient and incubated in optical 96-well reaction plates.

1.6 Quantitative Real Time PCR (qRT-PCR)

The quantitative real-time polymerase chain reaction (qRT-PCR) was performed using TaqMan Universal PCR Master Mix Kit (Thermo Fisher Scientific) according to the manufacturer's protocol and the equipment QuantumStudio3TM Real-Time PCR Systems. The reactions were performed for each patient and incubated in optical 96-well reaction plates. The thermocycling conditions were: 95 °C for 10 min, and 40 cycles of 15 s at 95 °C, followed by 1 min at 60 °C. After finalization of the qRT-PCR experiments, the average values of the cycle threshold (Ct) of the reactions in triplicate

were determined. The delta ct (Δct) method was adopted. The difference was calculated and plotted as follow: Ct total cycles 40 – Ct target. The difference was plotted as Δct directly.

1.7 In silico prediction of hsa-miRs target genes

In order to identify genes as target of hsa-miR-34, hsa-miR-181a, hsa-miR-150, hsa-miR-21, hsa-hsa-miR-155, hsa-miR-223, hsa-miR-125b and miR-146a linked to allergy and lung function we performed in silico analysis. The in silico identification of the target genes was performed using miR target link human (https://ccb-web.cs.uni-saarland.de/mirtargetlink/) and DIANA tools microT CDS (http://diana.imis.athena-innovation.gr/DianaTools/index.php?r=microT_CDS/index) databases. This latter database was used to check which miRNA target genes have already been validated experimentally for every miR tested.

1.8 Statistical analysis

All data are expressed as mean ± standard deviation (SD). We used both nominal (gender, co-morbidity, and treatment) and categorical (age, weight, and grade of disease) variables. The one-way ANOVA test was used to evaluate the differences between the groups. Differences identified by ANOVA were examined by using Kruskal-Wallis test followed by Dunn's Multiple Comparison Test. The Pearson test was used to evaluate the correlation between miRs expression and clinical characteristics (e.g. age and gender). GraphPad 8 software was used for the statistical analyses (GraphPad Software, San Diego, CA). The differences were considered significant for values of $p < 0.05$.

Supplementary Table S1. miRs are up- and down-regulated in PBMCs of asthmatic subjects compared to healthy controls

Asthma Profile	miRNA	Regulation in Asthmatics	Role/Pathway/Target
Dust mite induced severe asthma	miR-155	↑	Role: positively associated with the expression of the Th2 cytokines IL-5 and IL-13
			Pathway: allergic inflammation in T-cells

Bronchial asthma	miR-181a	↓	-
Severe asthma			
	miR-146a	↓	Pathway: vitamin D pathway in CD4+ T-cells
Dust mite induced asthma	miR-21-5p	↑	Role: predicted to inhibit differential responses to HDM in asthmatics versus non-sensitized controls
Th2, T helper 2; PBMC, peripheral blood mononuclear cell.			

Supplementary Table S2. microRNA concentration obtained from serum samples. Concentration of microRNA was measured on a Qubit 4 Fluorometer. Extraction efficiency is presented as the % of ath-miR-159a recovered during the isolation procedure (calculated using data from qRT-PCR analysis for ath-miR-159a in each sample versus the pure input used).

**% Extraction Efficiency**		
HNW, **Group A**	MANW, **Group B**	HNAO, **Group C**
83,27	97,05	91,05
82,25	85,18	88,96
84,78	85,94	83,57
82,37	82,48	89,99
85,70	81,15	90,06
84,84	82,78	
82,10	85,02	
82,75	82,70	
84,49	81,25	
85,85	82,00	
